# Fluorescence-guided detection of pituitary neuroendocrine tumor (PitNET) tissue during endoscopic transsphenoidal surgery available agents, their potential, and technical aspects

**DOI:** 10.1007/s11154-022-09718-9

**Published:** 2022-03-28

**Authors:** Rob A Vergeer, Robin E P Theunissen, Theodora van Elk, Iris Schmidt, Mark R Postma, Katalin Tamasi, J Marc C van Dijk, Jos M A Kuijlen

**Affiliations:** 1 Department of Neurosurgery, University of Groningen, University Medical Center Groningen, Groningen, The Netherlands; 2 Department of Endocrinology, University of Groningen, University Medical Center Groningen, Groningen, The Netherlands; 3 Department of Gastroenterology and Hepatology, University of Groningen, University Medical Center Groningen, Groningen, The Netherlands; 4 Department of Epidemiology, University of Groningen, University Medical Center Groningen, Groningen, The Netherlands

**Keywords:** Transsphenoidal surgery—pituitary adenoma—intraoperative fluorescence—fluorescence-guided surgery

## Abstract

Differentiation of pituitary neuroendocrine tumor (PitNET) tissue from surrounding normal tissue during surgery is challenging. A number of fluorescent agents is available for visualization of tissue discrepancy, with the potential of improving total tumor resection. This review evaluates the availability, clinical and technical applicability of the various fluorescent agents within the field of pituitary surgery. According to PRISMA guidelines, a systematic review was performed to identify reports describing results of *in vivo* application of fluorescent agents. In this review, 15 publications were included. Sodium Fluorescein (FNa) was considered in two studies. The first study reported noticeable fluorescence in adenoma tissue, the second demonstrated the strongest fluorescence in non-functioning pituitary adenomas. 5-Aminolevulinic acid (5-ALA) was investigated in three studies. One study compared laser-based optical biopsy system (OBS) with photo-diagnostic filter (PD) and found that the OBS was able to detect all microadenomas, even when MRI was negative. The second study retrospectively analyzed twelve pituitary adenomas and found only one positive for fluorescence. The third investigated fifteen pituitary adenomas of which one displayed vague fluorescence. Indocyanine green (ICG) was researched in four studies with variable results. Second-Window ICG yielded no significant difference between functioning and non-functioning adenomas in one study, while a second study displayed 4 times higher fluorescence in tumor tissue than in normal tissue. In three studies, OTL38 showed potential in non-functioning pituitary adenomas. At present, evidence for fluorescent agents to benefit total resection of PitNETs is lacking. OTL38 can potentially serve as a selective fluorescent agent in non-functioning pituitary adenomas in the near future.

**Supplementary information:**

The online version contains supplementary material available at 10.1007/s11154-022-09718-9.

## Introduction

Pituitary neuroendocrine tumors (PitNETs) are benign tumors arising from adenohypophyseal cells. They account for approximately 10–15% of all intracranial tumors and represent a heterogeneous group of tumors in their origin, growth patterns, and biological behavior [1–3]. PitNETs are divided into functioning and non-functioning. The latter are typically macroadenomas (≥ 10 mm) that generally present due to local space-occupying effects (optico-chiasmatic compression or cavernous sinus invasion), with or without hormone hyposecretion. Functioning PitNETs are mostly microadenomas (< 10 mm) and generally present with hypersecretory syndromes such as Cushing syndrome and acromegaly. Transsphenoidal surgery is the primary treatment of all PitNETs except for lactotroph tumors, which are primarily treated with dopamine agonists. Surgery removes the tumor mass and thereby decompresses vital structures. Total resection (TR) is particularly important in functioning pituitary adenomas to achieve endocrine remission (ER). Traditionally, differentiation between normal tissue and pituitary adenoma is made by the neurosurgeon based on macroscopic characteristics, e.g. tissue color and consistency. As such, ER for ACTH-producing adenomas is around 70%, and for GH-producing tumors 50–60% [4–6]. Therefore, more objective intraoperative tools can be valuable in the treatment of patients with functioning PitNETs.

Strategies to augment resection rates vary from measures that enhance preoperative diagnosis to improve visualization during surgery. Intraoperative fluorescence has been proven to enhance intraoperative differentiation and is currently applied in multiple surgical-oncology specialties such as gastrointestinal surgery, for applications as sentinel lymph node mapping, tumor detection, and tissue perfusion [7]. In 1948, a fluorophore named Sodium Fluorescein (FNa) was for the first time used in the field of neurosurgery for localization of brain tumors [8, 9]. Since then, fluorescence was further explored and is nowadays routinely used in other surgical-oncology specialties. In PitNET surgery, this imaging tool has not proven its value yet and therefore it is not part of the standard/routine surgical armentarium for removing PiTNET’s. The question remains if fluorescence guide surgery will be part of the routine surgical procedure for PitNET removal in future and thereby increasing TR and ER.

In the past years there has been an exponential growth in the publication of articles pertaining to fluorescence-guided surgery [10]. The aim of this review is therefore to identify reports describing the availability of fluorescent agents as well as the technical considerations.

1. Which fluorophores are currently being studied for use in the resection of PitNETs?
2. What is the mechanism of action of these fluorescent agents?
3. Which technical aspects are to be considered in the resection of PitNETs?
4. Does fluorescence-guided surgery provide a benefit to the resection of PitNETs in adults?

## Methods

Two separate systematic literature searches were conducted using PubMed, EmBase, and Cochrane (Table 1). Articles were reviewed in accordance with PRISMA guidelines. The search terms can be reviewed in table 1 in the appendix. All relevant articles until March 2021 were included in this review. The search resulted in 282 publications from PubMed, 25 publications from Embase, 17 publications from Google Scholar and the Cochrane database showed no publications. Duplicated, non-English, and abstract-only articles were excluded. Publications were reviewed for meeting inclusion criteria:

1. Regarding neurosurgery
2. Regarding adults
3. Regarding PitNET surgery
4. And/or regarding the technical aspects of fluorescent agent use
5. Use of fluorescent agents

**Fig. 1:**
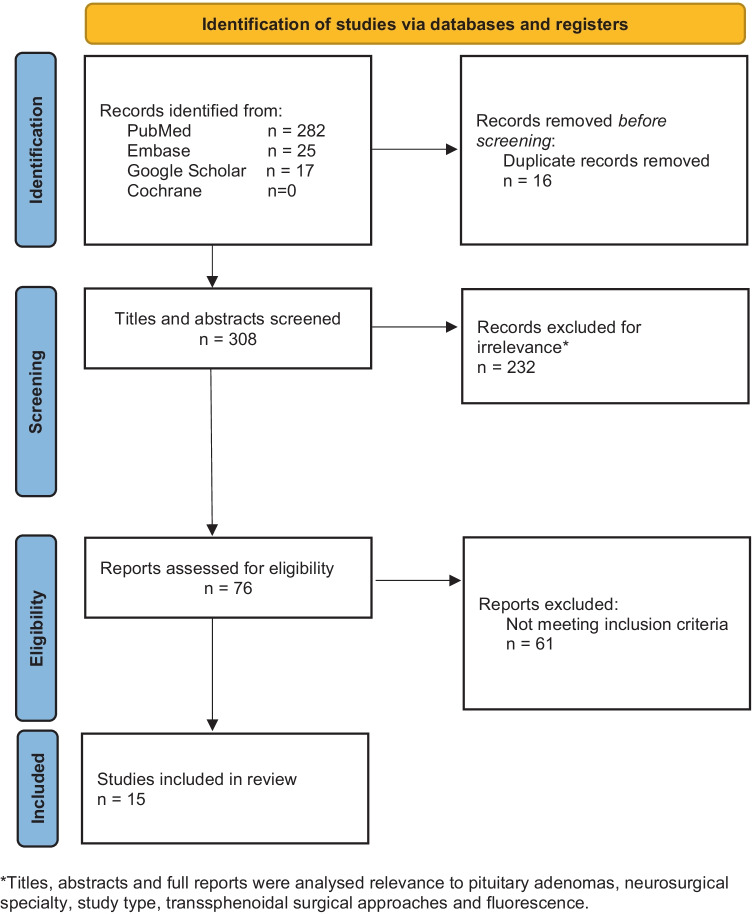
Study collection

Two reviewers (T.v.E. and R.E.P.T.) independently screened the titles and abstracts of all records identified. The full text of potentially eligible publications was retrieved and read for final selection. In case of disagreement, a third reviewer (R.A.V. or J.M.A.K.) was consulted to reach consensus. This process resulted in 15 publications on fluorescent agent use in PitNETs and on technical aspects considered relevant for the topic. See Fig. 1 for a flow diagram. The results are described in this systematic literature review.

**Table 1:**
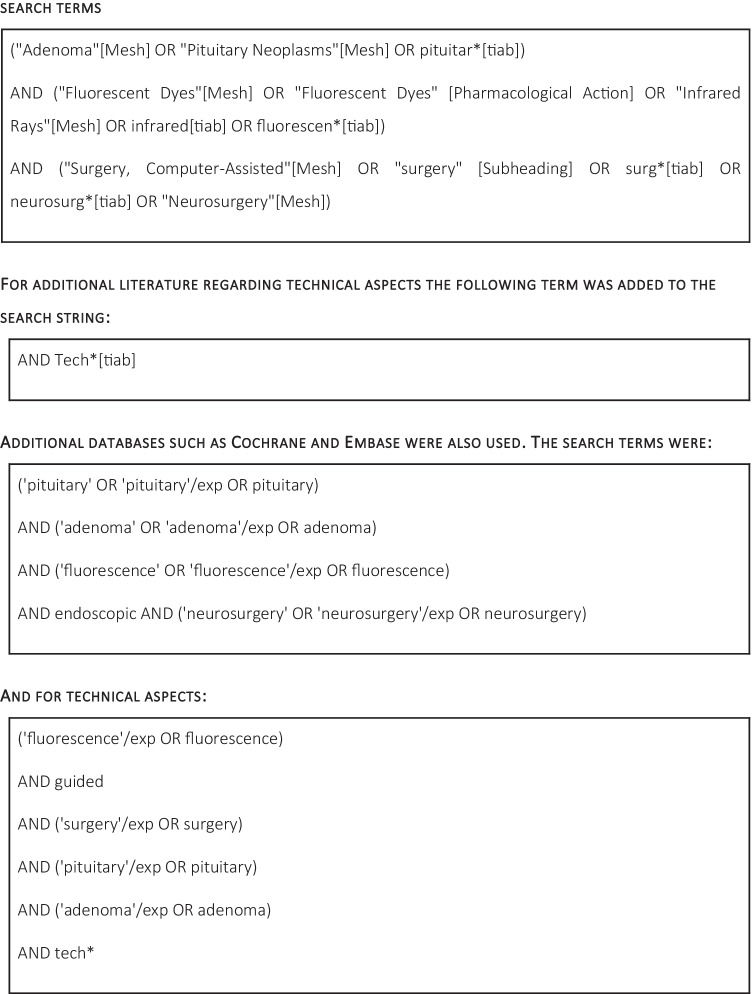
Search method

## Results

It is important to distinguish the various fluorophores in the way in which they act and how these fluorophores can be visualized. Fluorophores can be divided into 2 groups: (1) visible light fluorophores, e.g. Sodium Fluorescein and 5-ALA, that can be seen with the naked eye, thus precluding additional imaging instruments, and (2) Near Infrared (NIR) fluorophores, such as ICG and OTL38. To visualize NIR fluorophores additional imaging instruments, such as an infrared camera/filters, are needed (or a dedicated NIR imaging system).

### Sodium fluorescein

Sodium fluorescein (FNa) is an organic compound (C_20_H_12_O_5_) that functions as a dye. The light emitted from this yellow-green fluorophore is visible to the naked eye. FNa is used in the medical setting in ophthalmology for diagnostic use and in neurosurgery during the resection of high grade gliomas, metastatic lesions, treatment of cerebral aneurysms, and vascular malformations [11, 12]. FNa accumulation is the result of disturbed vascular permeability due to a disrupted blood–brain barrier [1].

In 2010, da Silva et al. performed a small observational FNa-study in skull base tumors(n = 6), which included only one PitNET. The authors reported a significant wavelength difference before and after FNa injection in all tumors [13].

In 2019, Romano-Feinholz et al. performed a pilot study to assess safety and feasibility of a hybrid technique using both endoscopy and microscopy in pituitary adenomas with intraoperative FNa, including 15 patients. Seven adenomas were non-functioning, six GH-secreting, one prolactin-secreting, and one ACTH-secreting. The authors compared pre- and postoperative MRI and categorized the extent of resection into total (100%), subtotal (80%-99%), and partial (< 80%). Intraoperative fluorescence was quantified with Adobe Photoshop using intraoperative images. Six tumors were resected totally, three sub-totally and six partially. Functioning adenomas were found to have the highest intensity of fluorescence (p = 0.003) [12].

**Table:** 

**Key points: Sodium Fluorescein (FNa)**
- The light emitted from this organic compound is visible to the naked eye
- Peak emission at 521 nm
- Used in ophthalmology and neurosurgery
- Functioning adenoma tumor tissue has the highest intensity of fluorescence
- Literature on FNa fluorescence in pituitary adenoma surgery is sparse and quality is low

### 5-ALA

5-ALA is a natural precursor for heme-synthesis. Cells metabolize ALA to heme in mitochondria. This pathway produces protoporphyrin-IX (PpIX). The conversion of PpIX to heme is rate-limiting in tumor cells, leading to accumulation of PpIX. PpIX is a photoactive molecule that absorbs violet-blue light and transmits in the red spectrum (600-650 nm) with peak emission at 635 nm [14, 15]. There are limitations to using 5-ALA. The auto-fluorescent characteristics of endogenous fluorophores such as lipofuscin and flavin lowers the signal-to-background ratio (SBR). Another disadvantage of 5-ALA is the low tissue penetration (micrometers) [16]. Also, a relative discomforting situation is that after administration patients have to be protected from sunlight and other ultraviolet radiation sources for 24 h.

Eljamel et al. studied 30 patients with pituitary adenomas; non-functioning macroadenomas (n = 14), gonadotrophin macroadenomas (n = 5), GH-secreting macroadenoma (n = 1), prolactinomas (n = 2), ACTH secreting microadenomas (n = 3), GH-secreting microadenoma (n = 1), and pituitary cysts (n = 4). Patients received 20 mg/kg body weight of 5-ALA, 3 h before surgery. Intraoperative they used a Photo-Diagnostic filter (PD) and a laser-based Optical Biopsy System (OBS). The OBS consisted of a Gallium nitride-laser unit emitting blue light at 440 nm and a compact spectrometer. Once the adenoma was localized, it was exposed and illuminated by violet-blue light (375–440 nm) using an endoscopic PD-system (Olympus® QTV55 and CLV-520, Germany) attached to an endoscope, and any fluorescent adenoma was removed until no further fluorescence was detectable. The sensitivity and specificity of the PD was found to be 80,8% and 75% respectively. The OBS sensitivity and specificity was found to be 95,5% and 100% respectively. PD showed a false negative rate of 19.2% and OBS 4.5%. The false positive rate for OBS was 0% versus 25% for PD. The OBS system was able to detect 100% of microadenomas, even when the MRI was negative [14].

Marbacher et al. retrospectively analyzed 458 cases with a variety of intracranial tumors. Positivity for 5-ALA was evaluated intermittently using the 440-nm blue-violet light source of a microscope (OPMI Pentero, Carl Zeiss). Twelve pituitary adenomas were assessed, but only 8% (1/12) displayed a positive fluorescence with 5-ALA. The fluorescence was evaluated by the surgeon. No further information on type of pituitary adenoma was available [17].

Micko et al. performed a multicenter retrospective study to investigate 5-ALA in endoscopic endonasal approaches. Twenty-eight patients with skull base pathologies were included and evaluated on their fluorescence status, quality, and homogeneity. The evaluation was done by both the surgical team and an external reviewer. Teams were blinded for each other’s results. Of the 28 pathologies, 15 were pituitary adenomas (seven functioning and eight non-functioning adenomas). No fluorescence was identified in all pituitary adenomas. Vague florescence was identified from the normal pituitary gland in a case of a gonadotroph macroadenoma, displayed vague fluorescence [18].

**Table:** 

**Key points 5-AminoLevulinic Acid (5-ALA)**
- 5-ALA is metabolized to PpIX and accumulates in tumor cells
- PpIX absorbs violet-blue light and transmits in the red spectrum of 600 nm to 650 nm with peak emission at 635 nm
- 5-ALA has been shown to improve glioma resection rates and progression-free survival rates
- Literature is sparse concerning 5-ALA usage during PitNET surgery. No strong added benefit for pituitary adenoma surgery has been proven yet

### Indocyanine green

#### Intraoperative bolus

Indocyanine green (ICG) is a near-infrared (NIR) fluorescent dye that has been approved by the Food and Drug Administration in 1959. Since then, it has been widely used in ophthalmology, reconstructive surgery, and abdominal surgery [19, 20]. In neurosurgery, ICG is utilized for intraoperative visualization of real-time cerebral perfusion. It is used for monitoring cerebral perfusion during surgery for neurovascular disorders [21]. Recently, the ICG-indication range of neurological pathology is broadened to gliomas, meningiomas, and pituitary adenomas [22, 23]. ICG is used as an IV bolus injection intraoperatively. In Second Window ICG, a term coined by Cho et al. ICG is administered as an infusion 24 h pre-operatively [16].

Litvack et al. performed an observational study in ten pituitary adenomas (five null-cell adenomas, one ACTH secreting adenoma, two GH secreting adenomas, one prolactin adenoma, and one mammosomatotroph adenoma). Fluorescence was analyzed with a photo diagnostic endoscope 45–90 s after ICG injection, before and after opening of the dura. A strong fluorescent nodule in the dura was noted, most likely due to hypervascularity by dural invasion of the adenoma. These fluorescent dural nodules were found in all patients with acromegaly, prolactinoma, and mammosomatotroph adenoma, but was not seen in null-cell adenomas (n = 5) that displayed lower fluorescence in tumor tissue versus higher fluorescence in pituitary gland tissue [24].

Sandow et al. performed an observational study in 22 patients. Thirteen patients were diagnosed with acromegaly, six with Cushing disease, and three with vision disorders or dizziness. In 11 patients, direct visualization of fluorescence in the lesion was noted, meaning that the adenoma displayed higher fluorescence than healthy tissue. The other 11 showed indirect visualization, in which the tissue surrounding the adenoma displayed higher fluorescence. All patients with Cushing disease displayed a higher ICG uptake than healthy tissue. It is interesting that all ACTH producing tumors (n = 6) were visualized directly, in comparison to the findings of Litvack et al. in which two hormone-producing adenomas displayed lower fluorescence than other adenomas. Apparently, timing of ICG injection affects fluorescence intensity, with a measured optimal fluorescence at 2.4 min after injection [23].

Verstegen et al. performed a clinical trial, including 10 patients with pituitary adenomas (four non-functioning, four ACTH-secreting, one GH-secreting, one prolactin-secreting tumor). Endoscopic transsphenoidal surgery was performed. Peroperatively 5 mg ICG was administered. All 10 patients displayed a minimum Fluorescence Contrast Ratio (FCR) of 1.3 (mean (SD) 1.5 ± (0.2)). FCR is calculated by dividing two regions of interest on a still image showing fluorescence of the adenoma and surrounding tissue. The authors noted that optimal FCR is generally considered to be > 2. They also noted that the timing of ICG is crucial for achieving optimal fluorescence [25].

Amano et al. studied 33 ICG bolus injections in 20 patients with a dosage of 6.25 or 12.5 mg/injection. Fifteen patients were diagnosed with a pituitary adenoma (nine GH-producing and six non-functioning adenomas). Two minutes post-injection, the posterior lobe was differentiated from the anterior lobe. After three minutes, the anterior lobe was fluorescent. The adenomas in the non-functioning group (n = 6) showed highest fluorescence after 1–7 min and gradually decreased, while the healthy tissue started to fluoresce 10–20 min post-injection. GH-producing adenomas showed fluorescence six minutes after injection. After debulking, the normal gland gradually became more fluorescent eight minutes post-injection. No growth hormone deficiencies were found post-operative. This is the first study that explored the extent of fluorescence versus time. These findings might explain the variable results in earlier studies using ICG [21].

**Table:** 

**Key points ICG**
- ICG is a near-infrared dye
- It has an excitation wavelength of 805 nm and emission in the 820-860 nm range
- Intra-operative dosages of ICG are timing sensitive for optimal results
- Administering multiple dosages is possible

#### Second window ICG (SWIG)

The mechanism of SWIG is based on the Enhanced Permeability Retention effect (EPR). EPR describes the enhanced permeability of tumor vasculature through which macromolecules can enter the tumor. It is hypothesized that ICG enters the cell through the EPR effect and is being retained in the cell. After a certain time period, healthy tissue has cleared ICG but tumor cells have become fluorescent by accumulation of ICG [15]. It is to be noted that ICG is not tumor specific, since disturbed permeability is seen in necrosis and inflammation as well.

Jeon et al. studied the use of SWIG on 8 patients (5 non-functioning adenomas, 1 somatotroph, and 3 corticotrope). ICG was administered 16–30 h before surgery. Pituitary adenomas demonstrated a signal-to-background (SBR) of 3.9 ± 0.8, which was 4 times higher than the fluorescence of surrounding tissue. While all 15 samples were positive for NIR signal, only 10 specimens were histopathologically proven adenoma tissue. The detection of false positive NIR signal, resulted in a decrease in specificity (20%). The sensitivity, PPV, and NPV of NIR-fluorescence were 100%, 71,43%, and 100%, respectively. The neurosurgeon was asked to evaluate intraoperatively whether the tissue was tumor or healthy tissue and was compared to histological findings. The sensitivity, specificity, PPV, and NPV of the neurosurgeon were 90%, 100%, 100%, and 83%, respectively. Jeon and colleagues suggested that a trained neurosurgeon in combination with NIR-fluorescence could lead to better resection results [26].

Cho et al. set up a study with two groups simultaneously. In the first, 16 patients received ICG 24 h pre-operatively (SWIG), while in the latter 23 patients received a folate analog, OTL-38. The SWIG-group consisted of seven non-functioning adenomas, four GH-secreting, two ACTH-secreting, one TSH-secreting, and 2 PRL-secreting adenomas. All 16 patients demonstrated NIR-fluorescence through the dura (SBR 2.7 ± 0.63). Upon opening of the dura and direct exposure of the tumor, the SBR increased to 4.1 ± 0.72. In addition, the pituitary adenomas were grouped by preoperative hormonal status. The hormonally active adenomas expressed a mean SBR of 4.1 ± 0.63, while in the non-functioning group an SBR of 4.0 ± 0.82 was detected. SWIG was 100% sensitive, but only 29% specific for neoplastic tissue, with a PPV of 82% and an NPV of 100% [16].

**Table:** 

**Key points Second Window ICG (SWIG)**
- SWIG mechanism is based on Enhanced Permeability Retention (EPR)
- PPV 71,43%—82%, NPV 100%
- Hormonal status of the adenoma has no effect on intraoperative fluorescence

### OLT38

OTL38 (On Target Laboratories, West Lafayette, Indiana) is a bioconjugate of a folate analog and a cyanine dye targeting folate receptor alpha (FRα) [27], which may be overexpressed in nonfunctioning pituitary adenomas [2]. A drawback of OTL38 is that FRα overexpression is not known before surgery, thus potentially resulting in significant false-negative results [27]. OTL38 has an excitation wavelength of 785 nm, and an emission wavelength spectrum of 800-835 nm, classifying OTL38 as NIR-fluorophore.

In a study performed by Lee et al. 15 patients with functioning or nonfunctioning adenomas were given OTL38. Six patients had non-functioning adenomas (four null-cell, one clinically silent gonadotroph, one fully silent somatotroph) and nine functioning adenomas (five corticotrope, three somatotroph, one somatocorticotrope). Three non-functioning PitNETs (50%) were found to overexpress FRα, while the functioning adenomas were found to have little to no FRα overexpression. Tumors with high FRα expression (n = 3) had an SBR 3.0 ± 0.29, while low FRα expression (n = 12) resulted in an SBR 1.6 ± 0.43 [28].

Remarkably, adenomas without overexpressed FRα should not have demonstrated fluorescence and yet did exhibit fluorescence. Thus, adenomas with little or no FRα expression showed an increased SBR as time passed because normal tissue cleared OTL38 faster than the tumor. The authors believe that these fluorescent adenomas showed an accumulation of OTL38, likely due to the tumors vasculature being leakier than healthy tissue, leading to enhanced permeability and retention effect in the tumor tissue, allowing passive accumulation.

The OTL38 patient subgroup of the mentioned study by Cho et al. [16] consisted of 23 patients (14 non-functioning adenomas, three GH-secreting, and six ACTH-secreting adenomas) and received OTL38 2–4 h preoperatively. All pituitary adenomas fluoresced with a mean SBR 1.6 ± 0.43 through the dura, and the mean NIR-SBR increased to 2.2 ± 0.88 upon tumor exposure. The 14 non-functioning adenomas showed a mean SBR 2.6 ± 0.91, the nine functioning adenomas a mean SBR 1.7 ± 0.47. When divided based on postoperatively determined FRα expression, the nine FRα-overexpressing adenomas demonstrated a mean SBR 3.2 ± 0.53. The 14 adenomas with low FRα expression had a mean SBR 1.6 ± 0.42. OTL38 had higher sensitivity and specificity when limited to the FRα-overexpressing adenomas, compared to assessment of all adenomas. Thus, OTL38 is highly sensitive and specific for pituitary adenomas that overexpress FRα but has limited utility in functioning adenomas.

Since OTL38 is folate receptor-alpha targeted (which is mainly expressed in non-functioning adenomas), the sensitivity and specificity was calculated only in non-functioning adenomas with (n = 28) and without (n = 18) overexpression of FRα. In the first, the sensitivity and specificity are 75% (51–90%) and 100% (60–100%) respectively, with PPV 100% (75–100%) and NPV 62% (43–77%). In the FRα overexpressing adenoma group (n = 18), the sensitivity and specificity are 100% (75–100%) and 100% (31–100%) respectively, with PPV 100% (75–100%) and NPV 100% (31–100%).

Another study by Cho et al. included 14 patients with non-functioning adenomas. Prior to opening of the dura, the group had a mean SBR 1.8 ± 0.40 that increased to 2.6 ± 0.92 after opening of the dura. The group was then divided into an overexpressing FRα and a non-FRα-overexpressing group. Nine adenomas overexpressed FRα with NIR-SBR 3.2 ± 0.52, whereas 5 non-FRα-overexpressing adenomas fluoresced with NIR-SBR 1.5 ± 0.21. Margin sample analysis demonstrated that the surgeon’s impression of the tissue had 83% sensitivity, 100% specificity, 100% PPV, and 89% NPV, while NIR-fluorescence demonstrated 100% for all values [3].

**Table:** 

**Key points OTL-38**
- OTL38 is a bioconjugate of a folate analog and a cyanine dye that targets folate receptor alpha (FRα), which is mainly overexpressed in nonfunctioning pituitary adenomas
- OTL38 has an excitation wavelength of 785 nm, and an emission wavelength spectrum of 800-835 nm, classifying OTL38 as a NIR-fluorophore
- Cho et al. demonstrated proof of concept and usability in non-functioning PitNETs pituitary adenoma

## Discussion

As gross total or total resection in PitNET surgery is around 70–80% [29, 30], there is a window of opportunity to raise the overall resection percentage. Particularly in functioning PitNETs, total resection is mandatory to cure the patient. Surgical aspects are of paramount importance in the general treatment of patients with PitNET. High-grade PitNETs are the most difficult to achieve total resection, since these tumors invade into the cavernous sinus with or without entanglement of the carotid artery. Especially for these complex tumors a supplemental diagnostic tool could be helpful in the intraoperative detection of residual tumor tissue. Therefore, we reviewed the literature to identify and value the various fluorescent agents specifically available in the context of endoscopic transsphenoidal surgery.

### Sodium fluorescein and 5-ALA

Sodium Fluorescein (FNa) and 5–ALA both belong to the group of ‘visible light’ fluorophores. 5-ALA has proven its feasibility in glioma surgery and FNa has an application in the detection of CSF leakage [31]. The application of FNa to aid in the resection of PitNETs is lacking evidence. The sparse results in the literature imply that FNa could be of use in the detection of functioning PitNETs but due to the limited evidence, the added value of FNa in PitNET surgery remains unclear. Eljamel et al. [14] concluded that 5-ALA might be of supportive value in the resection of the PitNETs. Specifically, in this study specialized hardware, such as a photo diagnostic filter and an Optical Biopsy System, was used to detect the 5-ALA fluorescence during surgery. In glioma surgery the violet/blue filter is integrated in the microscope, but a specific laser controlled Optical biopsy system is not present. Interesting to notice is that the OBS system was able to detect all of the microadenomas that were not seen on the MRI (6/6).

The evidence of 5-ALA as an additive diagnostic instrument for detection of residual pituitary tumor is not fully explored. Data is sparse and the described methods determining the fluorescence are limited and therefore difficult to reproduce. For certainly, more research will be needed to support 5-ALA as a suitable agent for detection of pituitary tumor tissue.

### Indocyanine green (ICG)

ICG demonstrated interesting results. A few studies demonstrated that non-functioning adenomas displayed a lower signal than the normal pituitary gland. Thus, indirect imaging of the tumor could be used to differentiate between normal tissue and tumor tissue [23, 24]. Furthermore, two small studies [23, 24] suggested that hormone producing tumors were visualized directly. However, this conclusion was not absolute as two hormone-producing adenomas displayed lower fluorescence [24]. Also in ICG, the described methods determining the fluorescence are limited and therefore difficult to reproduce. An explanation could be that timing of ICG injection and local tumor permeability are of key importance in relation to the optimal time frame of visualization and whether visualization is indirect or direct.

A technique that eliminates necessity for timing (time sensitiveness) is second window ICG (SWIG). Nevertheless, studies demonstrated that specificity of SWIG is low due to disturbed permeability. Since lower specificity leads to increased false-positive outcomes, this might be harmful as during surgery normal tissue could be falsely interpreted as tumor tissue. The SWIG study by Zhang et al. [27] demonstrated SBR to be 4 times higher in tumor than in surrounding tissue, but in histopathological examination not all fluorescent tissue could be classified as tumor tissue (false positive). In the study by Cho et al. [3], all PitNETs demonstrated NIR fluorescence, lacking significant difference in SWIG fluorescence signal between functioning and non-functioning PitNETs. As such, studies [16, 21, 23–26] seem to show contradictory results on ICG and SWIG application. However, SWIG has better results than intraoperative ICG bolus injection, considering the high NPV [16].

### OTL38

In studies on the use of OTL38, this fluorescent agent demonstrates its additive diagnostic use only in the population of non-functioning PitNETs. Histopathological analysis detected that mainly non-functioning PitNETs express the folate receptor alpha (FRα). Functioning PitNETs have none or less expression of FRα. Thus, OTL38 can only be applied in the removal of non-functioning PitNETS. Also, the factor time sensitivity played a role in the evaluation of the peri-operative results. PitNETs that did not overexpress FRα on histopathological analysis did exhibit fluorescence during surgery, because normal tissue cleared OTL38 faster than the tumor [28].

### Fluorescence guided surgery for endoscopic endonasal PitNET surgery; technical considerations

Understanding technical aspects of fluorescent imaging is crucial, especially within the sphenoidal cavity as its bony walls reflect the endoscopic light and blood remnants may disturb visibility, it is paramount to minimize all confounders that influence the detection quality of the fluorescent signal.

NIR fluorophores can emit their light through a thicker layer of tissue in comparison to visible light fluorophores, which is mainly absorbed by hemoglobin/blood. A second advantage is that NIR fluorophores are less influenced by the autofluorescence of nearby situated normal structures [3, 24, 28, 32]. On the other hand, the technique to visualize NIR fluorophores requires additional detection filters and hardware in comparison to visible light fluorophores. This can either be a microscope or endoscope with add-on NIR modules or a dedicated NIR imaging system. In a study by Cho et al. [3], the dedicated NIR-system showed superior SBR compared to the surgical microscope with NIR module. At the moment, dedicated NIR endoscopes do not have enough distinctiveness with respect to 2D HD 4 K endoscopes. Therefore, both NIR and 2D HD systems must be compared during surgery in order to define tumor tissue from non-tumorous tissue.

When using fluorescence during surgery, ideally only tumor tissue emits fluorescent signals, while other tissues such as healthy pituitary tissue should not. Thus, the detection of true positives is important. However, there are factors that can inhibit the detection of true positives. First, if there is a significant amount of fluorescence in the endonasal mucosa or dura surrounding the surgical field, the NIR camera may not properly detect smaller areas of fluorescence that could be tumor tissue. This can be combated by covering the surrounding area with surgical towels or gauze to isolate the surgical area, allowing smaller areas of residual fluorescence to be detected more easily [33]. However, this can be challenging in PitNET surgery. Second, care must be taken not to miss fluorescence hidden by overhanging tissue, necrosis, blood, or hemostatic adjuncts such as Surgicel® [33, 34]. The fluorophore must be directly excited by the laser. Thus, fluorophore concentrations hidden from direct light by overhanging structures, such as tumors that invade the cavernous sinus, can be easily missed. In one patient in a study by Verstegen et al. [25] intracavernous sinus bleeding prohibited assessment of the fluorescent signal of the adenoma tissue. Pooled blood can also obscure areas of NIR fluorescence by physically blocking excitation and emission light. Therefore, it is critical that hemostasis is achieved so that the tissue of interest can be placed in the direct line of the excitation source [33].

### Avoidance of false positivity

False positive signal is where healthy tissue produces a positive fluorescence signal. In order to detect tumor tissue alone, false positive signals need to be minimized. First, an important factor in the creation of false positives is the distance between the sensor and the tissue. Bringing the scope too proximal to the area of interest may result in artificially increased NIR fluorescence by flooding the area with excitation photons [32, 33]. Light decays exponentially as a factor of distance squared. Hence, when the scope is twice as close to the tissue, this results in a fluorescence signal that is 16 times greater, because the excitation light is 4 times stronger and 4 times as much emission is reaching the sensor. Multiple articles describe the use of the medial optico-carotid recess (MOCR) [3, 28, 32] as a useful landmark, maintaining the distance between the bilateral MOCR to be less than 50% of the overall field of view [32]. This offers a better discrimination between false-positive and true positive areas. In OT38 article [32], adenomas that did not overexpress FRα only fluoresced when the scope was brought into close proximity to the tumor, whereas adenomas that overexpressed FRα fluoresced strongly regardless of the scope distance.

Second, many NIR systems have variable gains that reflect the sensitivity of the camera sensor to emission photons. To be able to detect weaker signals a more sensitive camera is needed, and thus a higher gain can be applied. However, both signal and noise are enhanced, increasing the percentage of false positive signals. Many papers have described methods to minimize false positives caused by high gain settings. Cho et al. [33] describe a technique where the gain setting was preoperatively adjusted when the tumor was first exposed and maintained at that level throughout the rest of the surgery. Another technique is by Jeon et al. [26], where a positive control is used to set the gain. They dipped the control paper in an ICG solution and then waited till dry. An image was made from 12 mm away with an endoscope using both white light and NIR. The NIR detector was set to 50% gain, which was previously observed to provide a strong NIR signal without significant background noise. They noted that when using the automatic sensor gain adjustment feature inherent in the VisionSense camera software, true positive samples had a lower mean gain percent (76%) compared to that of false positive samples (97.5%). This suggests that the gain setting and thus the NIR sensor’s sensitivity was automatically increased to the point of creating false-positive fluorescence signals [26].

### The benefit of fluorescence-guided surgery in the resection of PitNETs

In general, fluorescence guided resection of PitNETs is not common practice. Most publications are proof of principle studies with small numbers of included patients and outcome is not related to clinical outcome, but have technical aspects of measuring and analyzing the fluorescent signals as the primary outcome parameters. Although test accuracy data (sensitivity, specificity NPV and PPV) presented in some studies show positive results we argue that interpretation of these figures must be done with great caution. It is questionable if these figures represent actual accuracy. We tried to verify, from the presented data, if these accuracy calculations were correct. In order to do so we contacted the respective authors by email to provide additional information, Unfortunately we could get no contact to date 1^st^ of October 2021.

The pitfall of the reviewed studies which presented accuracy data was that they included multiple samples per patient and provided no information as to which patient contributed to which sample, precluding the possibility for a patient-level analysis and an accurate estimation of confidence intervals. Disregarding the clustered nature of the data it could be especially problematic if the status of the samples originating from the same patient were highly correlated (e.g., sample A and sample B are more likely to be true positives if they were contributed by the same patient than by two different patients). Hence, the reported test characteristics (sensitivity, specificity, PPV, NPV) from studies that assume samples to be independent may not be representative of the patient-level test characteristics. Furthermore, in order to be able to generalize the results, it is important to consider the uncertainty of the estimates. Studies typically did only report the point estimate of test characteristics but no confidence intervals, which cannot be calculated without patient-level information. With the few data presented in several small cohorts it is impossible to draw any conclusion whether fluorescent supported surgery of PitNETs can lead to a higher track record of total resection and will be beneficial for patients with these tumors.

### Future perspectives

Currently, the use of fluorescent agents as a diagnostic during pituitary surgery is not standard care and most studies are proof of principle studies of non-targeted fluorophores. These fluorophores have their principal action through vascular permeability of the tumor, are non-targeted, and have more or less a narrow time window of imaging opportunity. In that perspective, there are many disadvantages. Nevertheless, the field of molecular biology is continuously developing and there is a high intensity quest for patient specific or tumor associated targets. Software, hardware, and high throughput analytical methods are available to unleash tumor associated markers in a short period of time.

Monoclonal antibodies such as bevacizumab (anti-VEGF) [35–37], girentuximab (anti carbonic anhydrase IX (CAIX) [38], cetuximab (anti EGFR) [39], and panitumumab (anti EGFR) [40, 41] are clinically approved oncolytic drugs that target specific cell membranous/cytoplasmatic bound antibodies and have been evaluated in clinical trials. Because the pharmacokinetic and pharmacodynamic properties of the monoclonal antibody and the NIR dye are already known from previous performed studies [42, 43], the combination of both can be constructed and evaluated [44].

## Conclusion

Fluorescent molecular endoscopy (FME) distinguishes from MRI, PET, and other radiological diagnostics in that it is real-time guidance in discriminating between healthy and diseased tissues. Properties of the imaging hardware and software can influence the images and can either limit or improve the ability to differentiate the targeted tissue from its surroundings. Interpretation of the endoscopic images requires comprehensive understanding of this technique, and the surgical team should carefully consider the limitations during the process of image-guided pituitary surgery. Attention must also be given to the accuracy and standardization of imaging systems and probes, in terms of clinical grade GMP production, quality control, and standard operating procedures related to stability [45].

Furthermore, special software and hardware must be facilitated in order to use FME during pituitary surgery. Therefore, it is to be expected that FME for pituitary surgery will initially be applied in high volume centers. For certain, further innovation on the field of FME in pituitary surgery will progress, but at this moment conventional endoscopic surgery still is the gold standard for maximum safe resection of pituitary tumors.

## Supplementary information

Below is the link to the electronic supplementary material.

Supplementary file1 (DOCX 31 KB)
